# SARS-CoV-2 specific antibody trajectories in mothers and infants over two months following maternal infection

**DOI:** 10.3389/fimmu.2022.1015002

**Published:** 2022-10-12

**Authors:** Melanie A. Martin, Monica Keith, Ryan M. Pace, Janet E. Williams, Sylvia H. Ley, Celestina Barbosa-Leiker, Beatrice Caffé, Caroline B. Smith, Amanda Kunkle, Kimberly A. Lackey, Alexandra D. Navarrete, Christina D. W. Pace, Alexandra C. Gogel, Dan T.A. Eisenberg, Bethaney D. Fehrenkamp, Mark A. McGuire, Michelle K. McGuire, Courtney L. Meehan, Eleanor Brindle

**Affiliations:** ^1^ Department of Anthropology, University of Washington, Seattle, WA, United States; ^2^ Center for Studies in Demography and Ecology, University of Washington, Seattle, WA, United States; ^3^ Margaret Ritchie School of Family and Consumer Sciences, University of Idaho, Moscow, ID, United States; ^4^ Department of Animal, Veterinary and Food Sciences, University of Idaho, Moscow, ID, United States; ^5^ Department of Epidemiology, Tulane University School of Public Health, New Orleans, LA, United States; ^6^ College of Nursing, Washington State University Health Sciences Spokane, Spokane, WA, United States; ^7^ Department of Anthropology, Washington State University, Pullman, WA, United States; ^8^ Elson S. Floyd College of Medicine, Washington State University, Spokane, WA, United States; ^9^ Washington, Wyoming, Alaska, Montana and Idaho (WWAMI) Medical Education, University of Idaho, Moscow, ID, United States; ^10^ Maternal, Newborn and Child Health & Nutrition, PATH, Seattle, WA, United States

**Keywords:** COVID-19, pediatric, IgG, IgA, infancy, breastfeeding

## Abstract

Infants exposed to caregivers infected with SARS-CoV-2 may have heightened infection risks relative to older children due to their more intensive care and feeding needs. However, there has been limited research on COVID-19 outcomes in exposed infants beyond the neonatal period. Between June 2020 – March 2021, we conducted interviews and collected capillary dried blood spots from 46 SARS-CoV-2 infected mothers and their infants (aged 1-36 months) for up to two months following maternal infection onset (COVID+ group, 87% breastfeeding). Comparative data were also collected from 26 breastfeeding mothers with no known SARS-CoV-2 infection or exposures (breastfeeding control group), and 11 mothers who tested SARS-CoV-2 negative after experiencing symptoms or close contact exposure (COVID- group, 73% breastfeeding). Dried blood spots were assayed for anti-SARS-CoV-2 S-RBD IgG and IgA positivity and anti-SARS-CoV-2 S1 + S2 IgG concentrations. Within the COVID+ group, the mean probability of seropositivity among infant samples was lower than that of corresponding maternal samples (0.54 and 0.87, respectively, for IgG; 0.33 and 0.85, respectively, for IgA), with likelihood of infant infection positively associated with the number of maternal symptoms and other household infections reported. COVID+ mothers reported a lower incidence of COVID-19 symptoms among their infants as compared to themselves and other household adults, and infants had similar PCR positivity rates as other household children. No samples returned by COVID- mothers or their infants tested antibody positive. Among the breastfeeding control group, 44% of mothers but none of their infants tested antibody positive in at least one sample. Results support previous research demonstrating minimal risks to infants following maternal COVID-19 infection, including for breastfeeding infants.

## Introduction

Most severe acute respiratory syndrome coronavirus 2 (SARS-CoV-2) infections in infants and children are asymptomatic or mild (1–3), and children have shown persistently lower rates of severe coronavirus disease 2019 (COVID-19) as compared to adults, even as the prevalence of pediatric cases increased over the course of pandemic (4). Children may have lower susceptibility to severe infection due to age-related immune differences (5), including pre-existing immunity from more frequent infection with other human coronaviruses (6–8); naïve immune responses that favor broad reactivity (9); lower rates of proinflammatory cytokine imbalances and related comorbidities (10); and reduced viral entry in the lungs, perhaps owing to more limited angiotensin converting enzyme 2 (ACE2) cell receptor expression or binding (10, 11).

Understanding factors associated with COVID-19 outcomes in infants and young children may help inform clinical therapies and policies regarding community transmission and mitigation strategies. However, research on infectious and immune outcomes within this age group has been limited, with the notable exception of research on fetal, perinatal, and neonatal transmission risks (e.g. 12–14). Beyond the neonatal period, infants may be at heighted risk of SARS-CoV-2 transmission from infected caregivers due to the frequency and intensity of close contact care and feeding (15, 16), and may have higher risks of severe infection relative to older children due to less developed immune responses (6, 17, 18). Outcomes of breastfed children exposed to SARS-CoV-2 infected mothers are of further interest, as milk is not a source of SARS-CoV-2 transmission but is a source of persistent SARS-CoV-2 specific antibodies (19–23) and likely other antiviral agents (24, 25). Therefore, additional research on antibody responses of infants and young children following maternal SARS-CoV-2 infection is needed to further delineate risks in this age group, and may be informative for families who continue to navigate infant and young childcare and feeding decisions following household infections or exposures.

We examined survey responses and antibody results assayed from capillary dried blood spots (DBS), which were collected prospectively from 83 U.S. mother-infant dyads (infants and young children aged 1 – 36 months, from here on referred to collectively as “infants”). Participants included SARS-CoV-2 infected mothers and their infants, as well as comparison groups of uninfected mothers and their infants. Most infants were breastfeeding during the study period. We compared household testing outcomes, symptoms, and maternal and infant anti-SARS-CoV-2 receptor binding domain (RBD) immunoglobulin (Ig) G and IgA responses across infected and uninfected groups and examined differences in trajectories of S1 and S2-reactive IgG concentrations over time among participants who exhibited SARS-CoV-2 seropositivity. Among infected mothers and their infants, we tested for differences in probabilities of maternal and infant IgG and IgA seropositivity, and examined selected maternal, infant, and household factors associated with likelihood of infant PCR or antibody positivity. To our knowledge, this is the first study to prospectively examine SARS-CoV-2 antibody responses in a sample of maternally exposed, mostly breastfed infants outside of the neonatal period.

## Materials and methods

### Study design and data collection

These analyses use survey data and capillary dried blood collected as part of a prospective, multi-site, repeated-measures, observational study conducted with U.S. mother-infant dyads between June 2020 and March 2021. The primary aims of the associated parent study were to (1) test for evidence of SARS-CoV-2 virus and antibodies in milk produced by acutely infected BF mothers (22, 23), and (2) to examine viral, antibody, and symptomatic outcomes in these dyads over time, as well as in comparison with those of non-BF infected mothers and their exposed infants, and never-infected BF mothers and infants. Participants were recruited through outreach by participating institutions (Tulane University; University of Idaho; University of Washington; Washington State University) and national social media advertising. Research personnel contacted prospective participants by phone to explain study procedures, determine eligibility, and obtain informed consent for all study procedures.

Participating mothers had to be ≥18 years of age and the caregiver of a child ≤36 months of age. The present study used only data from dyads enrolled after 28 days postpartum (antibody results of dyads enrolled < 28 days postpartum are described in the **Supplementary Information**). Additional eligibility requirements related to maternal COVID-19 status and child feeding status varied by recruitment group; no other demographic or health information were used as inclusion or exclusion criteria. Two primary study groups were recruited: (1) acutely infected breastfeeding and non-breastfeeding mothers who reported receiving a positive clinical polymerase chain reaction (PCR) test result within the past 7 days (COVID+, BF = 32; non-BF = 2); and (2) a control group of BF mothers who reported no known prior COVID-19 infections or exposures (BF control group, n = 26). To augment sampling, the present study also made use of survey and capillary dried blood samples collected opportunistically from COVID+ mothers who received a PCR-positive test result between 8 days and 2 months prior (BF n = 8, non-BF n = 4), and from mothers who enrolled while awaiting testing due to symptoms or close contact exposure, but subsequently tested PCR negative (COVID-, BF = 8, non-BF = 3). Surveys and samples collected from these latter groups were scheduled to best approximate the collection times of the parent COVID+ group up through two months following maternal infection. Positive and negative PCR results reported by mothers were not confirmed by additional in-house laboratory testing or follow-up. Data from all COVID+ mothers (regardless of time since positive PCR test and BF status) were combined (n dyads = 46) and compared with results from the COVID- and BF control groups. Recruitment groups and participant characteristics across the three study groups are described in **Figure S1** and **Table 1**.

**Table 1:** Characteristics of mothers and infants at enrollment, by enrollment group.

	COVID+ (n = 46)	BF control group (n = 26)	COVID- (n = 11)
	**Mean ± SD (range); % (n/n)**		
**Mothers**			
Breastfeeding	87% (40/46)	100% (26/26)	73% (8/11)
Age (years)	32 ± 4 (24 – 40)	32 ± 4 (26 – 38)	35 ± 7 (21 – 45)
Household size	4 ± 1 (3 – 7)	4 ± 1 (3 – 7)	4 ± 2 (3 – 8)
Completed education			
H.S./some college	29% (13/45)	24% (6/26)	0%
College degree	29% (13/45)	42% (11/26)	36% (4/11)
Graduate/professional	38% (17/45)	35% (9/26)	74% (7/11)
2019 Household income			
$20,000 – $49,000	21% (9/43)	24% (5/24)	30% (3/10)
$50,000 – $99,000	44% (19/43)	33% (8/24)	40% (4/10)
$100,000 or more	35% (14/43)	43% (11/24)	30% (3/10)
Ethnicity			
White non-Hispanic	85% (39/46)	84% (22/26)	91% (10/11)
White Hispanic	9% (4/46)	4% (1/26)	9% (1/11)
Non-white Hispanic	7% (3/46)	12% (3/26)	0%
Parity	2 ± 1 (1 – 4)	1.6 ± 0.9 (1 – 5)	2 ± 1 (1 – 4)
BMI*	27.7 ± 6.9 (18.0 – 47.5)	29.0 ± 8.6 (19.4 – 60.1)	28.4 ± 8.4 (17.8 – 42.9)
**Infants**			
Age (weeks)	36 ± 26 (4 – 132)	30 ± 20 (5 – 78)	67 ± 42 (11 – 149)
Sex			
Male	48% (22/46)	50% (13/26)	4 (36%)
Female	52% (24/46)	50% (13/26)	7 (64%)
Gestational weeks	39 ± 1 (36 – 42)	39 ± 2 (34 – 42)	39 ± 1 (36 – 40)
Birth mode			
Vaginal	64% (29/45)	85% (22/26)	82% (9/11)
Cesarean	35% (16/45)	15% (4/26)	18% (2/11)
Birth weight (kg)	3.5 ± 0.4 (1.9 – 4.3)	3.6 ± 0.5 (2.5 – 4.5)	3.4 ± 0.4 (2.7 – 4.2)
Birth length (cm)	50.9 ± 2.8 (43.2 - 58.4)	50.7 ± 2.3 (19.4 – 60.1)	50.6 ± 2.9 (45.7 – 55.9)

*BMI calculated from self-reported height and most recent weight.

Mothers answered survey questions by phone and collected capillary DBS from themselves and their infants over two months according to their enrollment group (**Table 2**) Surveys included questions about COVID-19 exposures, testing, and symptoms of all household members (see **Supplemental Information** for specific survey questions). Mothers were provided self-collection kits and instructions for finger and heel-prick capillary DBS for themselves and their infants using disposable safety lancets and HemaSpot™-HF filter paper devices (see **Supplemental Information** for instructions provided to participants). In families of four COVID+ and four COVID- mothers, other consenting household adults (n = 8) and children ages 2-5 years old (n = 4) provided HemaSpot DBS for antibody testing along with maternal and infant samples. **Table 3** summarizes DBS samples provided per enrollment group, participant category, and per collection time. Missing DBS data relative to survey data reflect preferences to opt-out of this procedure and difficulties collecting samples.

**Table 2:** Summary of data collection timeline by enrollment group.

Survey Day	COVID+/COVID-	BF Control Group
Day 0	Consent & demographic survey	Consent & demographic survey
Day 1	Interview (COVID-19 symptoms, testing, exposures, infant feeding)	Interview (COVID-19 symptoms, testing, exposures, infant feeding
Day 2-6	Follow-up interview	
Day 7	Follow-up interview **Blood spots**	Follow-up interview **Blood spots**
Day 14	Follow-up interview **Blood spots**	
Day 21	Follow-up interview **Blood spots**	Follow-up interview **Blood spots**
Day 30	Follow-up interview	
Day 60	Follow-up interview **Blood spots**	Follow-up interview **Blood spots**

**Table 3:** Count summary of dried bloods spots (DBS) analyzed per enrollment group, participant category, and time since maternal start date (first day of reported maternal symptoms or PCR test for COVID+/- groups, and days since enrollment date for BF control group).

Maternal group	Days 1- 13	Days 14 - 20	Days 21 - 30	Days 31 – 60+
	n = total # of participants (n = total DBS collected from those participants)			
COVID+	Mother = 13 (14) Infant = 7 (7) Other adult = 1 (1)	Mother = 24 (24) Infant = 13 (13) Other adult = 2 (2) Other child = 1 (1)	Mother = 27 (34) Infant = 18 (22) Other adult = 3 (4) Other child = 2 (3)	Mother = 29 (43) Infant = 19 (24) Other adult = 4 (7) Other child = 2 (3)
COVID-	Mother = 1 (1) Infant = 2 (2)	Mother = 5 (5) Infant = 4 (4) Other adult = 3 (3) Other child = 1 (1)	Mother = 4 (4) Infant = 4 (4) Other adult = 3 (3) Other child = 1 (1)	Mother = 5 (8) Infant = 5 (8) Other adult = 4 (7) Other child = 2 (4)
BF Control	Maternal = 21 (21) Infant = 13 (13)	Maternal = 10 (10) Infant = 6 (6)	Maternal = 11 (11) Infant = 10 (10)	Maternal = 20 (20) Infant = 15 (15)

All families in the COVID+ and COVID- groups were recruited between June and November 2020 before vaccines were widely available; dyads in the BF control group were recruited between November 2020 and January 2021. One COVID+ mother and nine mothers in the control group received at least one dose of a COVID-19 vaccine after beginning study participation. All data collection was completed by March 2021, before the SARS-CoV-2 delta and omicron variants were widely transmitted within the U.S.

### Laboratory analysis

Blood spot samples deemed to have sufficient sample quantity and quality (n = 363/380 collected) were analyzed for IgG and IgA antibodies targeting the SARS-CoV-2 RBD. Samples were analyzed using published enzyme immunoassay method for IgG (26), additionally modified to detect IgA, and validated for use with HemaSpot-HF DBS samples (see **Supplementary Information** for a detailed description of assay validation and protocols). Sample seropositivity for anti-S RBD IgG and IgA was determined relative to negative controls (ACCURUN Anti-SARS-CoV-2 Reference Material Kit, Series 2000, SeraCare), which were run in quadruplicate on all plates. Negative cutoff values for each plate were defined as the mean plus 3 standard deviations of the optical density for all four negative control wells. A signal to cutoff ratio (S/CO) was calculated for each sample by dividing the sample’s optical density by the cutoff value determined for each assay plate, and used to designate seropositivity: S/CO > 1 = positive, and S/CO < 1 = negative. The mean intraassay coefficients of variation (CV) for IgG and IgA were 8.5% and 12.6%, respectively; mean interassay CVs for low and high controls were: 5.4% and 11.5% (IgG), respectively, and 12.3% and 12.9% (IgA), respectively.

IgG positive samples with sufficient remaining volume (n = 125) were tested again using a quantitative multiplex assay for measurement of IgG antibodies to both the SARS-CoV-2 S1 and S2 subunits (Quansys Biosciences). Lower and upper limits of detection for S1 and S2 assays were 97.5 – 75,000 U/mL. Cutoff values for the quantitative IgG assay were determined according to the kit protocol, and all sample results quantitatively assayed were positive after adjusting for the dilution factor. Mean intraassay CVs for S1 and S2 were 8.4% and 6.3%, respectively. Mean interassay CVs for low and high controls were: 11.6% and 5.8% (S1), respectively; 9.5% and 5.4% (S2), respectively.

### Statistical analysis

For all enrollment groups and household member categories, we calculated prevalence of PCR testing, PCR positivity, COVID-19 symptoms in the two weeks following maternal start date (maternally reported for infants and other household members), and seropositivity (defined as at least one positive IgG or IgA antibody test result in any DBS collected). For the COVID+ and COVID- groups, maternal infection onset was defined as the date of onset of maternal COVID-like symptoms, or maternal PCR test date if asymptomatic. For the control group, maternal start date was the date of study enrollment. To examine group differences in IgG and IgA seropositivity over time, we plotted individual and mean IgG and IgA S/CO ratios for mothers and infants in each group and plotted mean IgG and IgA S/CO ratios from samples taken pre- and post-maternal vaccination in a subsample of the control group.

Differences in maternal and infant antibody responses were further examined among the COVID+ group only *via* a series of Bayesian models. All models were run in R v. 4.1.1 using the ‘brms’ package to fit linear and generalized linear models with full Bayesian inference in Stan (27–29). Models ran for 3000 iterations with 4 chains each, using the package’s default improper flat prior for population-level coefficients (29). Mean probabilities of IgG and IgA seropositivity (S/CO > 1) for maternal and infant samples were estimated with generalized linear models (Bernoulli distribution and logit parameterization), and reran with S/CO > 2 as the threshold for positivity as a measure of robustness. Infant PCR and IgG/IgA results were combined to examine the likelihood of any PCR/antibody positive test in association with infant age, total number of maternal symptoms reported following infection onset, and total number of other household members reporting positive PCR tests.

We then estimated differences in seropositivity probability among maternal and infant samples over time by fitting a non-linear model with a cubic-spline and interaction terms between participant category (mother vs. infant) and time since maternal infection onset (in days). Splines were fit with knot points at 14, 28, and 60 days for all IgG models and 14, 21, and 60 days for IgA models, given that RBD IgA concentrations appear to peak approximately 3 weeks after infection onset whereas IgG levels increase through week 4 (30). To examine quantitative differences in circulating antibody responses over time, we fit the same non-linear Bayesian models (with interaction terms for participant category and sample time) to IgG S1 and S2 concentrations. Correlations between S1 and S2 subunit concentrations were examined using the ‘rmcorr’ package for repeated measures correlations (31). All Bayesian model estimates have effective sample sizes greater than 1,000, and Markov chain Monte Carlo (MCMC) chains across all models showed sufficient convergence (all Rhat=1.00).

## Results

### Symptoms and household testing

Participants in our study experienced relatively mild COVID-19, with no mothers or infants hospitalized for this disease. In the first two weeks following maternal start date, 87% of COVID+ mothers reported experiencing at least one systematically surveyed COVID-19 symptom (**Table 4**). COVID+ mothers most commonly reported fatigue (65%), loss of smell or taste (59%), congestion or runny nose (57%), headache (54%), and cough (44%) (**Table 4**). In contrast, all COVID- mothers but only 20% of mothers in the BF control group reported any COVID-19-like symptoms during the first two weeks of suspected infection or enrollment. Similar proportions of infants displaying any COVID-19-like signs were reported for the COVID+ group (50%), COVID- group (64%), and the BF control group (42%). Infant COVID-19-like signs were also more diverse, with only congestion/runny nose reported for more than 20% of infants in the COVID+ group (**Table 4**).

**Table 4:** Systematically surveyed COVID-19 symptoms/signs reported by mothers and for their infants during the first two weeks following maternal infection onset or enrollment date (for COVID+/- and BF control groups, respectively); N/A = not asked of that group; (-) = none reporting.

Symptom	COVID+ (n = 46)		COVID- (n = 11)		BF Control Group (n = 26)	
	Mothers	Infants	Mothers	Infants	Mothers	Infants
Cough	44% (20)	17% (8)	46% (6)	18% (2)	–	8% (2)
Fever	15% (7)	15% (7)	18% (2)	9% (1)	–	–
Fatigue	65% (30)	N/A	72% (8)	N/A	12% (3)	N/A
Difficulty breathing	22% (10)	2% (1)	36% (4)	–	–	–
Diarrhea	26% (12)	15% (7)	36% (4)	–	–	8% (2)
Sore throat	33% (15)	–	46% (5)	–	–	–
Congestion/runny nose	57% (26)	37% (17)	91% (10)	55% (6)	12% (3)	27% (7)
Headache	54% (25)	–	64% (7)	–	4% (1)	–
Red/itchy/watery eyes	2% (1)	7% (3)	27% (3)	9% (1)	8% (2)	–
Sneezing	15% (7)	17% (8)	18% (2)	27% (3)	4% (1)	12% (3)
Stomach pain	11% (5)	2% (1)	–	–	4% (1)	8% (2)
Loss of smell/taste	59% (27)	N/A	–	N/A	–	–
Vomiting	N/A	2% (1)	N/A	–	–	–
Low reactivity	N/A	9% (4)	N/A	9% (1)	–	–
Rash on stomach/trunk	N/A	2% (1)	N/A	9% (1)	–	–
Reduced feeding/nursing	N/A	11% (5)	N/A	27% (3)	–	4% (1)
Any symptoms	87% (40)	50% (23)	100% (11)	64% (7)	20% (5)	42% (11)

Among households of COVID+ mothers, the proportion of infants demonstrating any symptoms in the two weeks following maternal infection onset was lower than that reported for other adults and children aged 5-9 years, but double the rate reported for other children under 5 years of age years (**Table 5**). Rates of PCR testing were lower in infants compared to other child age groups, and positivity rates across all children tested were lower than those of other household adults (**Table 5**). Due to the nonuniform testing within families, it was not possible to determine index cases or estimate average household secondary attack rates. Rates of PCR testing across age groups were similar among households of COVID+ and COVID- mothers.

**Table 5:** COVID-19 PCR testing, symptom, and antibody testing results for mothers, infants, and other household members by enrollment group.

	PCR tested	PCR+*	COVID-19 symptoms**	Antibody+*
**COVID+ (n = 46)**				
Mothers	100% (46/46)	100% (46/46)	87% (40/46)	89% (32/36)
Infants	41% (19/46)	42% (8/19)	50% (23/46)	48% (12/25)
Other household children (< 5 years)	64% (14/22)	29% (4/14)	23% (5/22)	50% (1/2)
Other household children (5 - 9 years)	53% (9/17)	44% (4/9)	53% (9/17)	N/A
Other household adults (18+ years)	87% (45/52)	62% (28/45)	60% (28/47)	50% (4/4)
**BF control group (n = 26)**				
Mothers (unvaccinated)	0%	N/A	28% (5/18)	47% (8/17)
Infants (unvaccinated mothers)	0%	N/A	44% (8/18)	0% (0/13)
Mothers (vaccinated)	0%	N/A	0% (0/8)	83% (5/6)
Infants (vaccinated mothers)	0%	N/A	38% (3/8)	20% (1/5)
Other household children (< 5 years)	0%	N/A	14% (1/7)	N/A
Other household children (5 - 9 years)	0%	N/A	0% (0/7)	N/A
Other household adults (18+ years)	19% (5/27)	0% (0/5)	7% (2/31)	N/A
**COVID- (n = 11)**				
Mothers	100% (11/11)	0% (0/11)	100% (11/11)	0% (0/6)
Infants	45% (5/11)	20% (1/5)	64% (7/11)	0% (0/7)
Other household children (< 5 years)	50% (1/2)	0% (0/1)	50% (1/2)	50% (1/2)
Other household children (5 - 9 years)	33% (2/6)	50% (1/2)	50% (3/3)	N/A
Other household adults (18+ years)	50% (7/14)	50% (3/6)	54% (7/13)	100% (4/4)

*PCR+ defined as reporting a positive PCR test within 14 days following maternal symptom onset or test date; Antibody+ defined as providing at least one blood sample that tested IgG or IgA positive. Differences in PCR and antibody sample sizes within groups reflect differences in reported PCR testing vs. capillary blood samples provided for antibody testing. Antibody results for other household children and adults were collected opportunistically from a small subset of families.

**Mothers reported or observed any COVID-19 symptoms during the first two weeks following maternal infection onset (for COVID-19_pos_ and COVID-19_neg_ groups) or the first week following study enrollment (for BF control group).

### Differences in antibody responses among COVID+, COVID-, and control groups

**Figure 1:**
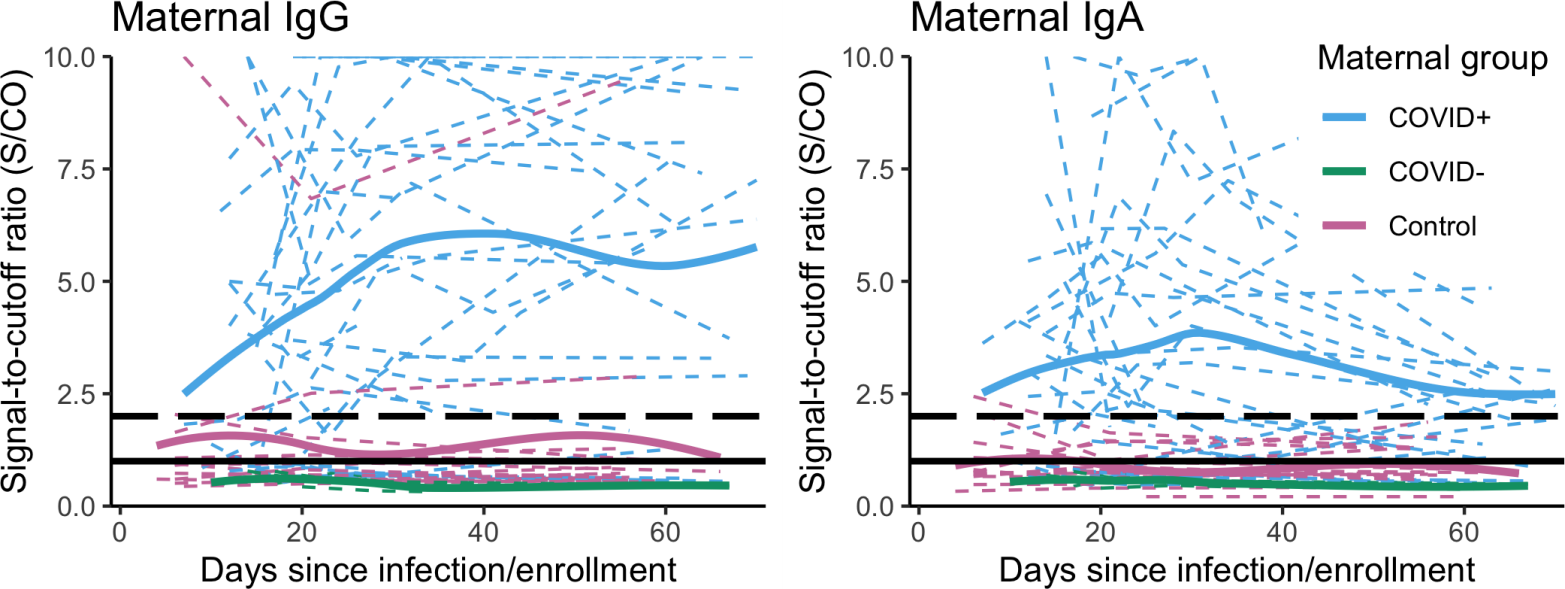
Individual maternal IgG and IgA S/CO trajectories (dashed, colored lines) over two months following maternal infection onset or enrollment, and loess smooth curves (solid, colored lines) fit for each enrollment group: COVID+ (n = 36, n_obs_ = 116); COVID- (n = 6, n_obs_ = 18); BF control group, (n = 20, n_obs_ = 50, excluding samples collected post-vaccination). S/CO=1.0 (solid black line), S/CO=2.0 (dashed black line). Y-axis is truncated at S/CO = 10; samples with S/CO between 10-20 not shown (IgG n_subj_ = 12, n_obs_ = 19; IgA n_subj_ = 6, n_obs_ = 4).

**Figure 2:**
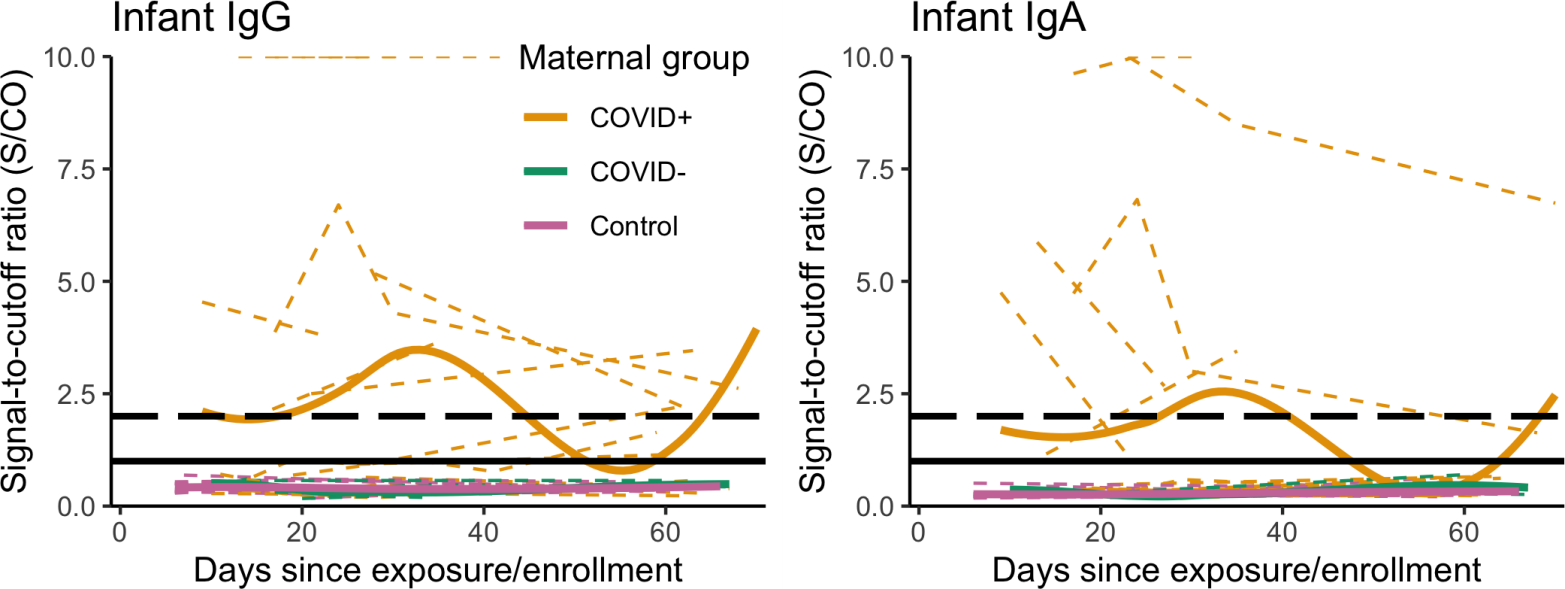
Individual infant IgG and IgA S/CO trajectories (dashed, colored lines) with group-level loess smoothes (solid, colored lines) over two months following maternal infection onset or enrollment, by maternal study group: COVID+ (n = 25, n_obs_ = 66); COVID- (n = 7, n_obs_ = 18); and BF control group, excluding samples collected post-maternal vaccination (n = 15, n_obs_ = 35), S/CO=1.0 (solid black line), S/CO=2.0 (dashed black line). Y-axis is truncated at S/CO = 10; samples with S/CO between 10-20 not shown (IgG n_subj_ = 3, n_obs_ = 11; IgA n_subj_ = 2, n_obs_ = 3).

Nearly all (96%) blood spot samples collected in this study (363/380) yielded sufficient blood for in-house IgG and IgA testing (maternal samples: n = 195; infant samples: n = 128; opportunistically collected samples from other household children and adults: n = 40. The majority of COVID+ mothers (36/46) provided at least one DBS sample with sufficient blood for antibody testing (median samples/subject = 4), with 89% (32/36) of these mothers testing positive for IgG or IgA at least once (**Table 5**). In contrast, DBS samples were collected from just 25/46 of their exposed infants (median samples/subject = 2), with 48% (12/25) testing positive for IgG or IgA (**Table 5**). Combined PCR and antibody test results were available for only 11 infants in the COVID+ group: 7/11 had concordant results (three PCR+/antibody+ and four PCR-/antibody-) and 4/11 had discordant results (three PCR-/antibody+, one PCR+/antibody-). Individual and mean signal-to-cutoff (S/CO) trajectories plotted for COVID+ dyads demonstrate generally weaker signal and greater number of infant samples falling below the S/CO > 1 positivity threshold as compared to maternal samples (**Figures 1**, **2**).

No samples collected from COVID- mothers (n subjects = 6/11, n samples = 18) or their infants (n subjects = 7/11, n samples = 18) tested IgG or IgA positive (**Table 5**, **Figures 1**, **2**). This included four samples collected from one infant reported to test PCR+, and all maternal and infant samples (n = 25 samples) collected from four families in which samples provided concurrently by fathers tested antibody positive (data not shown). Among dyads in the BF control group, DBS samples were returned by 23/26 mothers and 18/26 of infants, with 6/23 mothers receiving at least one COVID-19 vaccination following enrollment. Unexpectedly, 47% (8/17) of the non-vaccinated control group mothers returned at least one sample that tested IgG or IgA positive (18/46 samples total, **Figure 1**). Six of these mothers returned positive samples at the first DBS collection seven days after enrollment (**Table 2**), while two mothers who initially tested negative returned positive samples beginning at 21 days after enrollment. As demonstrated by individual and mean S/CO trajectories, IgG and IgA antibody responses of these mothers were generally weaker as compared to those of COVID+ mothers, and none of their corresponding infant samples tested IgG or IgA positive (**Figures 1**, **2**).

Among the vaccinated mothers in the BF control group, 5/6 tested antibody positive at least once post-vaccination. The plotted mean IgG and IgA S/CO trajectories for maternal samples in this group peak at 4-6 weeks post-vaccination, with a similarly weaker IgA signal as observed in COVID+ mothers (**Figure 2**, **Figure S2**). Maternal samples that did not test IgG positive were collected between 30 days prior to and 5 days after the first vaccine dose (**Figure S2**). The plotted mean S/CO trajectories from corresponding infant samples do not cross the positivity threshold for IgG or IgA (**Figure S2**), although one infant in this group tested antibody positive in samples taken 6 and 62 days after the mother’s first vaccine dose.

### Antibody responses of COVID+ mothers and exposed infants

**Figure 3:**
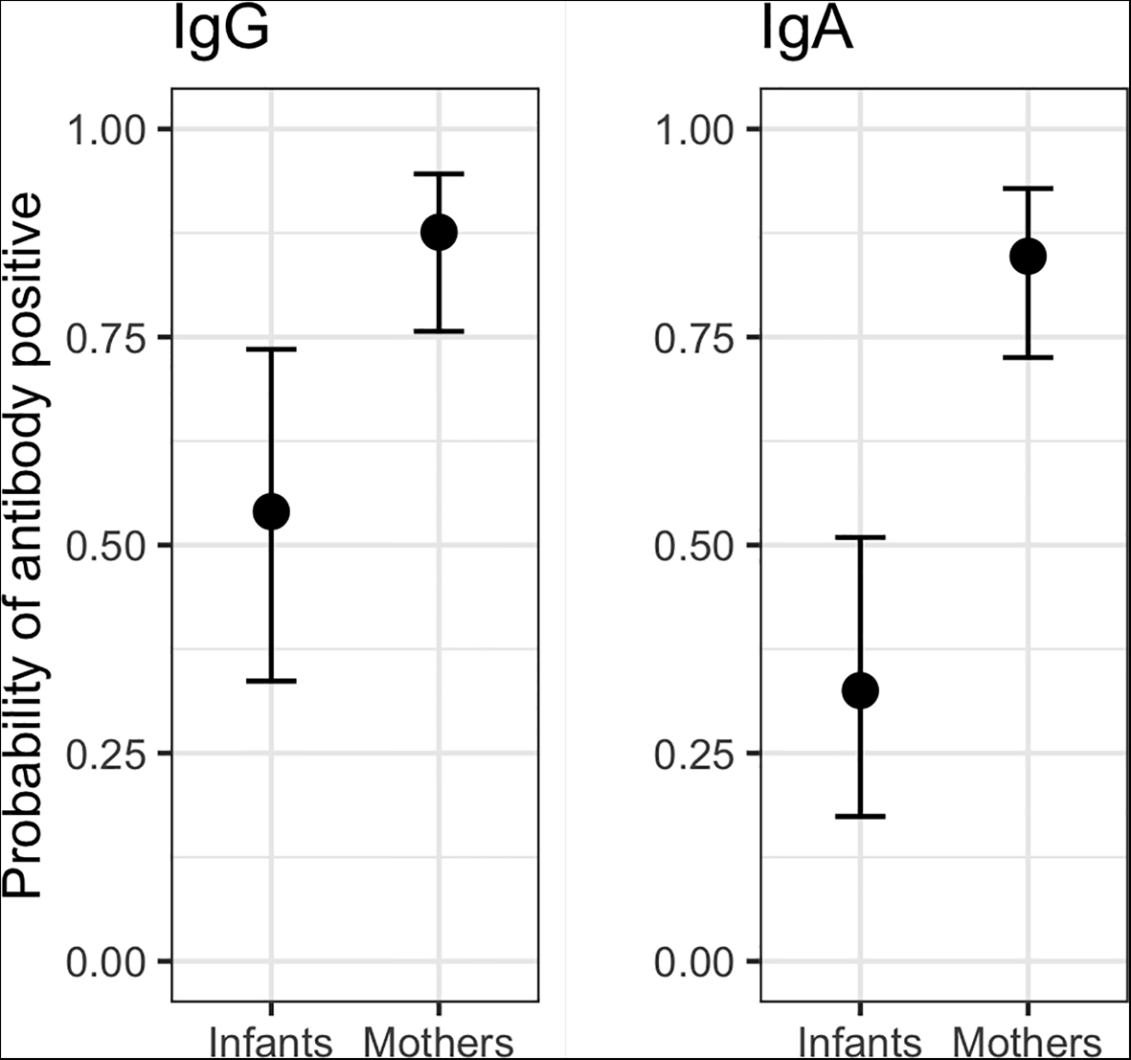
Predicted mean probability (with 95% credible intervals) of IgG and IgA seropositivity across all samples collected from COVID+ mothers (n = 36, n_obs_ = 116) and their exposed infants (n = 25, n_obs_ = 66). IgG infant = 0.54 [0.33 – 0.74], IgG maternal = 0.87 [0.76 – 0.95]. IgA infant = 0.33 [0.17 – 0.52]; IgA maternal = 0.85 [0.72 – 0.93].

**Figure 4:**
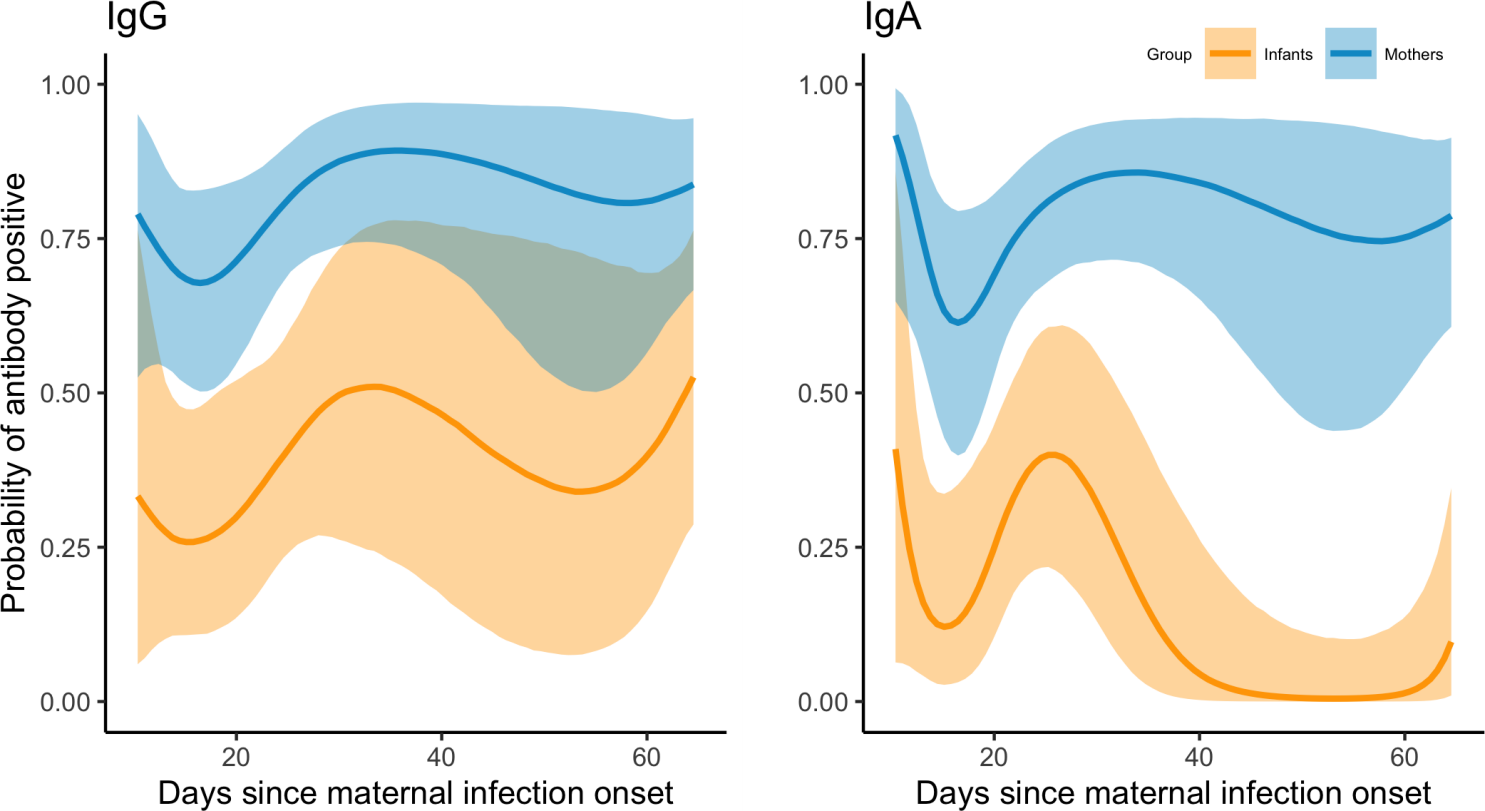
Mean estimated probabilities (95% credible intervals) of IgG and IgA seropositivity (S/CO ≥1) in samples collected from COVID+ mothers (n = 36, n_obs_ = 116) and their exposed infants (n = 25, n_obs_ = 66) in the two months following maternal infection onset.

Mean probabilities of IgG and IgA seropositivity (S/CO > 1) were lower for infants than mothers in the COVID+ group. However, infant estimates also show greater uncertainty and a higher mean probability of IgG compared to IgA seropositivity (**Figure 3**). For both maternal and infant samples, estimated mean probabilities of IgG and IgA seropositivity increased between approximately 15-20 days after maternal infection onset and thereafter remained elevated, but with greater uncertainty (**Figure 4**). Mean probability of IgA seropositivity showed a similar pattern for maternal samples but decreased in infant samples approximately 30 days after maternal infection onset (**Figure 4**). Applying a higher threshold of seropositivity (S/CO > 2) did not substantially alter mean probability estimates for infant or maternal IgG (infant est. w/95% CI: 0.49 [0.29 – 0.69]; maternal: 0.84 [0.71 – 0.93]) but resulted in lower probability estimates for IgA (infant: 0.22 [0.11 – 0.38]; maternal: 0.61 [0.44 – 0.75], **Figure S3**).

Due to limited antibody and PCR testing in infants of COVID+ mothers, we also examined factors associated with infant positivity defined as ever testing positive by PCR or IgG/IgA testing. In univariate models, infant positivity was associated with total maternal symptoms reported at infection onset and with the total number of other household members testing positive, but was not associated with infant age, presence/absence of maternal cough, total number of other household members reporting COVID-19 symptoms, or the ratio of total household members testing positive to total tested (**Table 6**). In a multivariate model including both total maternal symptoms and other household members infected, effect estimates of both variables were mediated by adjusting for the other (**Table 6**). Model fit and performance, as indicated by leave-one-out-cross-validation information criterion and Pareto k estimates, were also poorer than those of respective univariate models.

**Table 6:** Factors associated with probability of COVID-19 positivity infants of COVID+ mothers (positivity defined as ever testing positive by PCR or IgG/IgA antibody testing).

Univariate models	Est. w/95% CI
Age of infant at enrollment (months)	0.15 (-0.78, 1.17)
Total maternal COVID-19 symptoms at onset	**0.58 (0.18, 1.08)**
Maternal cough at onset (ref. = “no”)	1.59 (-0.02, 3.37)
Total other household members testing positive	**2.07 (0.48, 4.15)**
Total other household members symptomatic	0.94 (-0.00, 2.04)
Total ratio household positive/household tested	1.36 (-0.62, 3.36)
**Multivariate model**	
Total maternal COVID-19 symptoms at onset	0.42 (-0.06, 0.97)
Total other household members testing positive	**1.96 (0.24, 4.19)**

Coefficient estimates with 95% Confidence Interval.

Significant associations are in bold.

**Figure 5:**
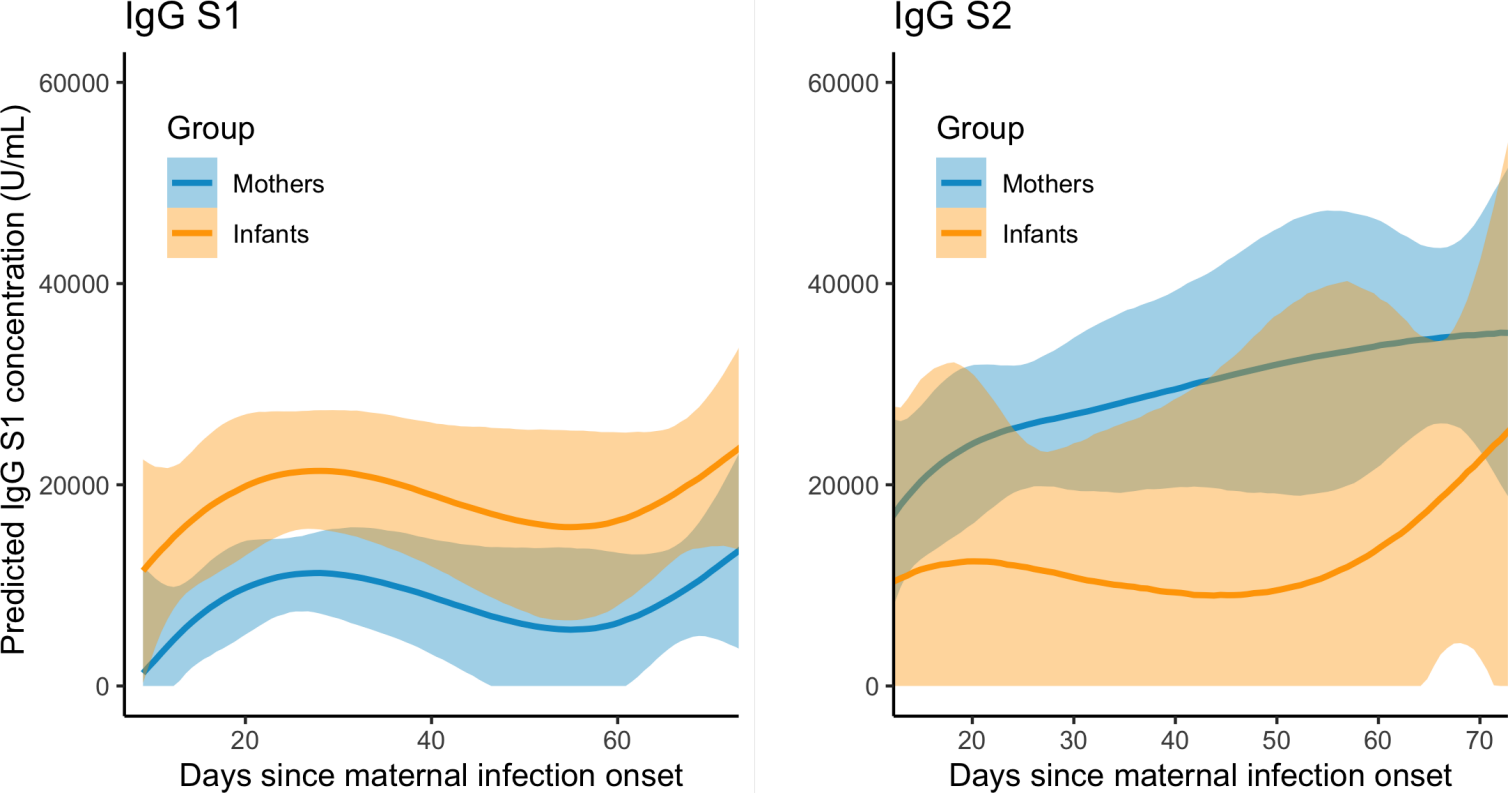
Predicted IgG S1 and S2 concentration (with 95% credible intervals) in COVID+ mothers (n = 26, n_obs_= 66) and exposed infants (n = 9, n_obs_=17) over two months following maternal infection onset.

Among the COVID+ mothers and infants who tested IgG positive, and adjusting for time since maternal infection onset, we observed a trend of higher estimated mean subunit S1-reactive IgG concentrations in infants as compared to mothers (S1 infant est. w/95% CI = 20616 [14176 – 27059] U/mL; maternal est. = 10508 [5398 – 15888] U/mL). S2 concentrations were in infant samples (S2 infant est. = 13376 [3253 – 23127] U/mL; maternal est. = 27157 [19244 – 35042] U/mL) but overlapped substantially with credible intervals of maternal samples, and in some cases spanned biologically implausible negative values (**Figure 5**). The pattern of higher IgG S1 and lower IgG S2 in pediatrics vs. adult samples persisted when combining maternal and infant samples with all positive IgG samples collected opportunistically from other household adults and children, though again credible intervals were large and overlapping (**Figure S4**).

In repeated measures correlations, IgG S1 and S2 concentrations were moderately correlated in maternal samples (r = 0.43 [0.11 – 0.75], df=48, p =0.002), but could not be accurately estimated for infant samples due to the limited sample size. In Bayesian linear model estimates adjusting for day of sample collection, the total number of maternal COVID-19 symptoms reported at infection onset was positively associated with IgG S2 (1968 [189 – 3727] U/mL), but not IgG S1 concentrations (-2.90 [-804 – 836] U/mL). The presence or absence of COVID-19 symptoms in infants during the first two weeks following maternal infection onset was not associated with infant IgG S1 or S2 concentrations, with wide credible intervals spanning biologically implausible values (S1: 22775 [-14096 – 60562] U/mL; S2: 14579 [-9088 – 38029] U/mL).

## Discussion

In this study of SARS-CoV-2 infected mothers and their infants (nearly 90% of whom were breastfeeding), infants were considerably less likely than mothers to exhibit IgG and IgA seropositivity. In other studies of adults and children, SARS-CoV-2 seropositivity is strongly associated with concurrent household infection (15, 32), and symptom severity is associated with higher viral load (33) and higher household secondary attack rates (34, 35). Similarly, in our study, probability of infant positivity (as determined by PCR or antibody testing) was associated with the total number of other household members testing positive and the total number of maternal symptoms reported following maternal infection onset. Mean probabilities of IgG and IgA seropositivity remained relatively constant in mothers in the two months following maternal infection onset, but declined for infants, particularly for IgA. However, uncertainty among infant estimates was markedly greater and increased over time, likely a result of more limited sampling, mild symptoms, and faster rate of decline in antibody responses.

Previous studies have observed that SARS-CoV-2 RBD-IgG and IgA levels in adults begin to rise during the first days of infection, peak at 15-28 days post-symptom onset, and then remain detectable for at least 6 months post-infection (36–39). In children and adults, asymptomatic and mild COVID-19 is associated with lower antibody levels and earlier declines in antibody levels (5, 40–42). Children have been observed to exhibit greater SARS-CoV-2 cross-reactivity in humoral responses, with robust spike-specific antibody responses lasting at least 12 months, but weaker and more quickly waning responses to nucleocapsid (7) and reduced neutralizing activity (42). In general, however, adults and children appear to have similar viral, cellular, and serological responses in cases of asymptomatic or mild COVID-19, with immune responses only diverging with severe disease (5). Lower probability estimates of IgA relative to IgG seropositivity in our study may reflect lower assay sensitivity in combination with earlier peaks and declines in IgA (30), particularly among infants who had milder symptoms.

Among COVID+ dyads who tested IgG positive, we observed a higher mean S1-reactive IgG concentration in infants as compared to mothers, and no difference in S2 concentrations. This pattern persisted when examining all available positive samples from children and adults. In previous studies conducted with uninfected, pre-pandemic blood donors, SARS-CoV-2 S2-reactive IgG antibodies have been observed at higher prevalence in children as compared to adults (8), but with no S2 cross-reactivity observed in infants less than 1 year old, and little cross-reactivity in S1 across ages (43). The S2 domain of the spike protein is more conserved and more likely to elicit coronavirus cross-reactivity compared to S1 (44). Higher pre-existing SARS-CoV-2 S2-reactive antibody concentrations in children have been suggested to result from their more frequent human coronavirus exposures, which could confer enhanced cross-reactive immunological memory and therefore greater protection against severe infection (7, 8). The marked uncertainty and lower mean S2-reactive IgG concentration among positive infant samples in this study may reflect age-related differences in immune development, as well as a relative lack of recent human coronavirus exposures due to pandemic-related changes in social interactions during the time of data collection (June 2020 to March 2021).

Within households of the COVID+ group, rates of PCR-positivity and symptoms were lower among infants and other household children as compared to mothers and other household adults. These results concur with findings from global studies conducted during the first year of the pandemic, in which infection rates were lowest among infants and young children in communities (32, 45) and households (46–48), with household secondary attack rates ranging from 5% to 55% and exhibiting marked variation across age groups (34, 49–52). In an observational study of SARS-CoV-2 infected children, infants < 2 years of age were observed to have lower viral loads but lower prevalence of symptomatic cases as compared to children 2 years and older (53). We observed a slightly higher prevalence of PCR positivity and symptomatic cases among infants as compared to other household children aged 2-5 years in our study, though differences in PCR positivity may reflect age-related differences in exposures related to care and feeding, testing, and parental perception of symptoms and risks that influenced testing. PCR positivity rates across all infants and children in our study may be underestimated due to limited testing at the time of data collection, as later seroprevalence studies demonstrated much higher rates of infection in children that were often asymptomatic (15).

Interestingly, while no COVID- mothers or their infants tested antibody positive, nearly half of non-vaccinated mothers in the BF control group tested antibody positive despite no known prior COVID-19 infection or exposures. These samples were collected from November 2020 to March 2021, by which time community transmission had increased in many areas. Thus, these mothers may have been unknowingly asymptomatically infected prior to or shortly after enrolling. No concurrently collected infant samples tested antibody positive, however, suggesting that these infants were either not infected following maternal exposure, or experienced very mild infections with quickly waning antibody responses.

Our study is limited by the small sample size and relatively few infants with concurrent PCR test and antibody test results available for comparison. PCR test results were maternally reported and not confirmed by additional follow-up or in-house laboratory testing, which may increase error in our results due to possibility of false positive and negative test results among participants. Many of the participants in our study were still adhering to community physical distancing mandates or recommendations at the time of data collection. We also relied on participant-collected DBS from finger- and heel-prick capillary samples as part of no-contact data collection protocols developed to ensure participant and researcher safety. Antibody titers obtained from capillary and venipuncture samples are equivalent (54), and community seroprevalence testing of SARS-CoV-2 using self-collected DBS samples has been previously validated (55). To further ease participant burden, our home-collection DBS kits included HemaSpot devices, which require less blood and are more easily handled than standard filter paper cards. Hemaspot DBS with sufficient sample remaining for qualitative antibody testing were collected from 78% and 54% of COVID+ mothers and their infants, respectively, and from 88% and 73% of mothers and infants, respectively, in the BF control group, suggesting that capillary blood spot collection was more challenging for infants and particularly within families experiencing acute infections. Despite these limitations, our study provides additional support for the feasibility of DBS self-collection to expand SARS-CoV-2 seroprevalence testing with targeted study groups.

To date, the majority of studies on paired maternal-infant COVID-19 outcomes have examined evidence related to vertical transmission *in utero*, during delivery, or through breast milk. Such studies have been critical in establishing low risks of vertical transmission in comparison with other exposures, and have bolstered recommendations for continued maternal-infant contact, rooming-in and breastfeeding (56–60). Research has also conclusively established that milk of SARS-CoV-2 infected and vaccinated mothers is a source of long-lasting antibodies with neutralizing activity against SARS-CoV-2 (21–23, 61, 62). However, limited research has examined maternal-infant infectious outcomes in later infancy, or in relation to specific caregiver exposures or behaviors that influence infant infection risks.

To our knowledge, this is the first study to prospectively examine SARS-CoV-2 antibody responses in infants (most of whom were breastfeeding) following maternal infection outside of the neonatal period (> 28 days). Limited survey research has demonstrated a similarly low rate (32%) of reported PCR positivity in breastfed infants of COVID-19 positive mothers (60). Elsewhere, non-infected breastfed infants were observed to exhibit elevated SARS-CoV-2 specific salivary IgA two months after maternal COVID-19 infection, without concurrent elevation of serum antibodies, suggesting direct stimulation of infant salivary antibodies from antigen-antibody immune complexes in milk (63). Still, it remains unclear how the timing of maternal infection or vaccination, total household infectious burden, and the frequency and type of milk feeding may further mitigate infant outcomes of infected lactating mothers. While we were unable to evaluate any direct protective effect of breastfeeding on infant outcomes in our study, exposed breastfed infants did not appear to be at higher risk of SARS-CoV-2 infection as compared to other (non-breastfed) household children and adults. Our findings further support previous research demonstrating minimal risks to infants following maternal COVID-19 infection, including for breastfeeding infants.

## Data availability statement

The data presented in the study are available as a dataset on figshare: https://doi.org/10.6084/m9.figshare.20422245.v3.

## Ethics statement

The studies involving human participants were reviewed and approved by the institutional review boards of the University of Idaho (20-056, 20-060), the University of Washington (STUDY00010215), and Tulane University (2020-602). Adults provided written and verbal consent for themselves and their minor children to participate in this study. The patients/participants provided their written informed consent to participate in this study.

## Author contributions

Concept and design: MAM, EB, RP, JW, CM, SL, CB-L, BF, MMc, and MKM. Acquisition, analysis, or interpretation of data: All authors. Drafting of manuscript: MAM. Critical revision of the manuscript for important intellectual content: All authors. Statistical analysis: MK and MAM. Obtained funding: MAM, EB, RP, JW, SL, CB-L, DE, MMc, MKM, and CM. Administrative, technical, or material support: MAM, EB, RP, JW, SL, CB-L, MMc, MKM, and CM. Supervision: MAM, RP, JW, SL, CB-L, MMc, MKM, and CM. All authors contributed to the article and approved the submitted version.

## Funding

This work was supported by the National Science Foundation (IOS-BIO 2031753, 2031715, 2031888, 2031761) and the Bill and Melinda Gates Foundation (INV-016943). Additional support for this work was provided by the University of Washington Center for Studies in Demography and Ecology (NICHD P2C HD042828), the University of Washington Department of Anthropology, US National Institutes of Health (R01 HD092297-03), Washington State University Health Equity Research Center, USDA National Institute of Food and Agriculture (Hatch project IDA01643). The funding agencies had no role in the collection, management, analysis, and interpretation of the data; preparation, review, or approval of the manuscript; and decision to submit the manuscript for publication.

## Acknowledgments

We sincerely thank the participating families in this study for volunteering their time and considerable efforts made to self-collect blood samples.

## Conflict of interest

KL is employed by Traverse Science.

The remaining authors declare that the research was conducted in the absence of any commercial or financial relationships that could be construed as a potential conflict of interest.

## Publisher’s note

All claims expressed in this article are solely those of the authors and do not necessarily represent those of their affiliated organizations, or those of the publisher, the editors and the reviewers. Any product that may be evaluated in this article, or claim that may be made by its manufacturer, is not guaranteed or endorsed by the publisher.
