# Supplementary material for: SARS-CoV-2 specific antibody trajectories in mothers and infants over two months following maternal infection
Source: Front Immunol. 2022 Oct 12;13:1015002. doi: 10.3389/fimmu.2022.1015002 (PMC9596287; doi:10.3389/fimmu.2022.1015002)
Supplement: Supplementary file 1 [file DataSheet_1.docx]

Supplementary Material

# Supplementary Text

## Survey questions and Hemaspot DBS home collection instructions

A PDF file containing survey questions analyzed in this study, as well instructions for home Hemaspot DBS collection is available at [10.6084/m9.figshare.21171811](https://doi.org/10.6084/m9.figshare.21171811)

## Validation of in-house qualitative IgG and IgA assays with HemaSpot dried blood samples

IgG antibodies to the receptor binding domain (RBD) of the SARS-CoV-2 spike protein were measured using an anti-SARS-CoV-2 RBD IgG assay method described by Stadlbauer et al. (2020). Minor modifications were made to create a version of the assay to detect anti-SARS-CoV-2 IgA antibodies. The protocols for both antibody subtypes were adapted for use with samples collected on a HemaSpot device, and evaluated using paired serum and contrived HemaSpot samples with known antibody status.

Assay plates were coated with 2 µg/mL of recombinant RBD (RayBiotech cat# 230-30162) in phosphate buffered saline (PBS; Fisher BP2438) added at 50 µL/well and incubated overnight at 4˚C. The next day, plates were washed with PBS containing 0.1% v/v Tween 20., and a solution of the same PBS-Tween 20 (PBS-T) with 3% w/v dry milk powder (AmericanBio #AB10109-01000) added was used to block the plates (200 µL/well, 2 hours at room temperature [RT]). After decanting the block solution, prepared controls and samples were added at 100 µL/well and incubated for 2 hours at RT. Plates were then washed again before adding secondary antibodies labeled with horseradish peroxidase (HRP). For the anti-SARS-CoV-2 IgG assay, the secondary antibody was an HRP-labeled anti-human IgG (Fab‐specific) secondary antibody produced in goats (Sigma #A0293), and for the anti- SARS-CoV-2 IgA assay the secondary antibody was an HRP-labeled, cross-adsorbed goat-anti-human IgA (Invitrogen A18787). Both secondary antibodies were diluted in PBS-T with 1% w/v dry milk to dilutions determined by titration (1:3000 for IgG, 1:2000 for IgA) and added at 100 µL/well and incubated for 1 hour at RT. After a final wash, an OPD chromogen solution (SigmaFast OPD, Sigma-Aldrich #P9187, prepared according to the supplied instructions) was added and color was allowed to develop for 10 minutes before the reaction was stopped with the addition of 3M HCl. The optical density of the color reaction (492nm test and 620nm reference) was quantified using a BioTek microtiter plate reader and data reduction was performed using Gen5 software. Positive and negative controls purchased from SeraCare (ACCURUN Anti-SARS-CoV-2 Reference Material Kit, Series 2000) were run in quadruplicate wells on every plate. Negative ACCURUN controls were used for calculating cutoff values: the cutoff value was defined as the mean plus 3 standard deviations (SD) of the optical density for the four negative control wells. A signal to cutoff ratio (S/CO) was calculated by dividing the optical density value for each sample by the cutoff value; an S/CO above 2 was considered positive, S/CO of 1 to 2 was weak positive, and S/CO below 1 was negative.

To adapt the assay for use with HemaSpot samples, positive and negative SeraCare ACCURUN controls were prepared by combining washed red blood cells from a donor without SARS-CoV-2 antibodies with positive and negative control serum purchased from SeraCare (ACCURUN Anti-SARS-CoV-2 Reference Material Kit, Series 2000). The contrived whole blood positive and negative cutoff controls were dropped onto HemaSpot devices to create positive and negative HemaSpot controls that were run in duplicate on every plate along with the original SeraCare serum controls in liquid form. The same method was used to create paired serum and HemaSpot samples from a panel of 15 anti-SARS-CoV-2 antibody positive serum specimens purchased from BioIVT (part # HMSRM-COVIDREC); blood specimens in the panel were collected 1 to 46 days following diagnosis with COVID 19 (average 25 days). Samples were screened using a qualitative multiplex anti-SARS-CoV-2 IgG assay (Quansys Biosciences 691649HU) to confirm they were antibody positive. The panel of 15 serum specimens was then tested using the IgG and IgA in-house assays, and subsets were selected from the BioIVT panel to create high and low titer pools for both IgG and IgA, with each pool containing serum from 4 different donors. The pooled serum was combined with washed red blood cells and used to prepare semiquantitative HemaSpot controls (IgG high and low and IgA high and low) that were run in duplicate on every assay plate.

The day before assay, HemaSpot cutoff controls, semiquantitative controls, and samples were eluted from the HemaSpots in PBS-T. One blade was removed from the HemaSpot with forceps and placed in an elution plate, then 175 µL of PBS-T was added to submerge the blade. The volume of serum equivalent contained in each HemaSpot blade was estimated at 7 µL by prior experimentation in our lab with quantitative protein assays (unpublished data not shown), yielding an estimated dilution of 1:25 for the resulting elutes. After allowing the blades to elute overnight at 4˚C, the 1:25 eluates were combined with an equal volume of PBS-T with 1% w/v dry milk to produce an estimated final dilution of 1:50. Serum versions of all samples and controls and were diluted to 1:50 in PBS-T with 1% w/v dry milk.

The panel of 15 antibody positive samples purchased from BioIVT was used to evaluate concordance between the antibody levels in HemaSpot and serum samples. Matching pairs of each sample type were tested on the same assay plate, and optical density results were compared. For the IgG assay, the mean ± SD optical density of the HemaSpot results was 88% ±14% of the optical density of the matching serum sample; for IgA HemaSpot results were 82% ± 12% of matching serum results. Values from the two sample types were highly correlated for both assays (R^2^ 0.974 for IgG and 0.961 for IgA, n = 15 pairs).

Study specimens collected using the HemaSpot device were tested using the elution and assay methods described above, with a single elution used for both IgG and IgA. A single person assigned all the HemaSpots a subjective score for saturation of the blades ranging from 1, completely filled, to 5, no useful sample. When the blades were not completely filled, multiple blades were used to reach a total of approximately 7 µL of sample volume to achieve a final dilution close to 1:50 (32% of samples). The cutoff value for each assay was calculated using the liquid SeraCare ACCURUN negative control results. Assays were considered valid if both the liquid and HemaSpot versions of the SeraCare reference materials produced the expected results (positive or negative), and if the semiquantitative HemaSpot positive controls (IgG high and IgG low; IgA high and IgA low) produced the expected relative optical density values (i.e. higher optical density for the high versus the low control). S/CO was used as a semiquantitative measure of antibody concentration in the study specimens. The dose-response relationship is expected to be non-linear because the color signal becomes saturated at very high antibody concentrations.

References:

Stadlbauer, D., Amanat, F., Chromikova, V., Jiang, K., Strohmeier, S., Arunkumar, G. A., Tan, J., Bhavsar, D., Capuano, C., Kirkpatrick, E., Meade, P., Brito, R. N., Teo, C., McMahon, M., Simon, V., & Krammer, F. (2020). SARS-CoV-2 seroconversion in humans: A detailed protocol for a serological assay, antigen production, and test setup. *Current Protocols in Microbiology*, 57, e100. doi: [10.1002/cpmc.100](https://doi.org/10.1002/cpmc.100)

## Quantitative IgG assay

Samples that were positive or weakly positive for IgG in the in-house assay, and for which the HemaSpot quality score indicated properly filled blades allowing more precise estimates of the sample volume, were tested again using a quantitative multiplex anti-SARS-CoV-2 IgG assay against spike glycoprotein 1 (amino acids 1-674 of subunit 1) and spike glycoprotein 2 (amino acids 685-1211 of spike subunit 2) capture proteins along with negative and positive control spots in the same plate well (Quansys Biosciences #711649HU). Hemaspots were eluted to a dilution of 1:25 as described above, then the eluates were diluted further in kit diluent to a final dilution of 1:75. Cutoff values were determined according to the kit protocol. All sample results were positive after adjusting for the dilution factor.

## Antibody results from excluded participants

Three COVID+ mothers were enrolled before 28 days postpartum and confirmed or likely to have been exposed during delivery. For one dyad, all maternal and infant samples collected over the first 45 days postpartum tested positive, while a single infant sample collected at 80 days postpartum tested negative. For another dyad, maternal samples collected at 24-38 days postpartum tested antibody positive while a sample collected at 73 days postpartum tested negative. A single infant sample from this dyad collected at 24 days postpartum tested negative. The last of these dyads included two concurrently lactating mothers and their newborn. The birth mother was exposed in the hospital; the partner and infant tested PCR-positive approximately two weeks later. All DBS samples collected from both mothers and the infant tested positive across the two-month observation window.

Antibody analyses also excluded one maternal and one infant sample of a COVID+ dyad collected 22 days after the mother received a 1^st^ vaccine dose (66 days following maternal infection onset). All maternal (n = 4) and infant samples (n = 4) collected from this dyad tested IgG/IgA positive. However, between the samples collected pre- and post-vaccination, maternal IgG S/CO ratios increased five-fold (with slight increases in IgA S/CO), IgG S1 concentrations doubled, and S2 increased from 19,437 UI/mL to beyond the limit of detection. Conversely, IgG and IgA S/CO ratios decreased between the infant samples collected before and after maternal vaccination (there was insufficient sample for IgG S1 & S2 assaying of the post-vaccination sample).

# Supplementary Figures

**Supplementary Figure 1.**  Study recruitment groups described by maternal COVID-19 status and child feeding status


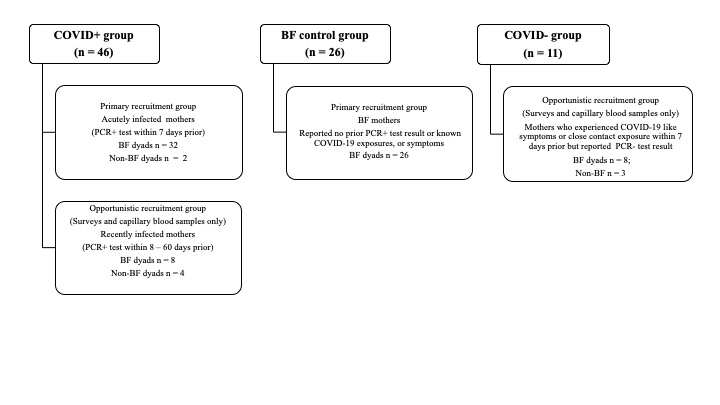


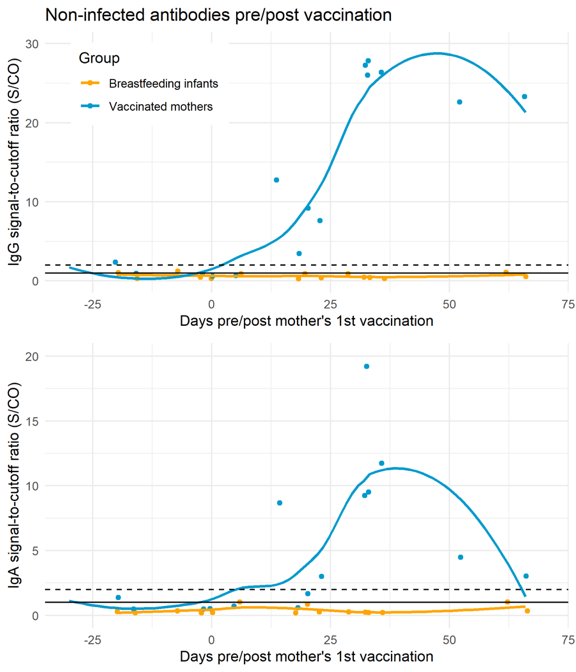
**Supplementary Figure 2.** Loess smoothes of IgG (top) and IgA (bottom) S/CO before and after (two months) first vaccine dose among vaccinated mothers in the BF control group (n=6, n_obs_=16) and their infants (n=6, n_obs_=12); S/CO=1.0 (solid black line), S/CO=2.0 (dashed black line)

**Supplementary Figure 3.**


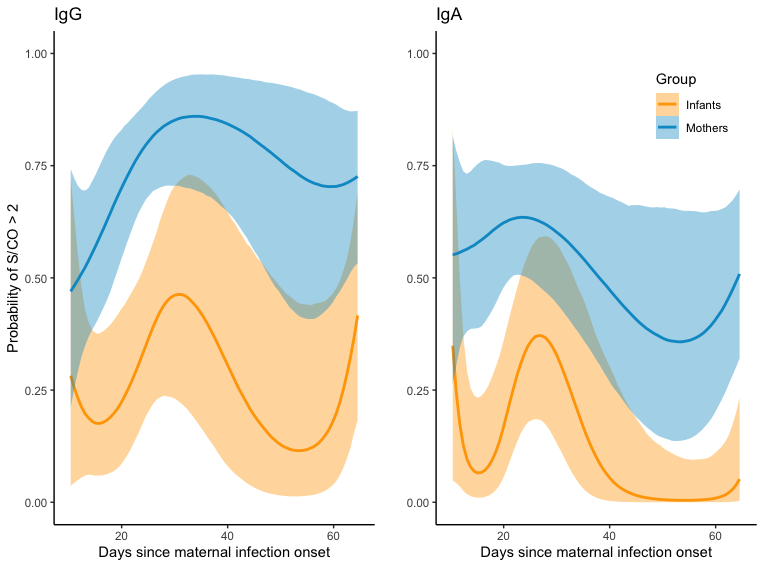
Mean estimated probabilities (95% credible intervals) of IgG/IgA seropositivity at higher positivity threshold (S/CO > 2) in samples collected from COVID+ mothers (n = 36, n_obs_ = 116) and their exposed infants (n = 25, n_obs_ = 66) in the two months following maternal infection onset

**
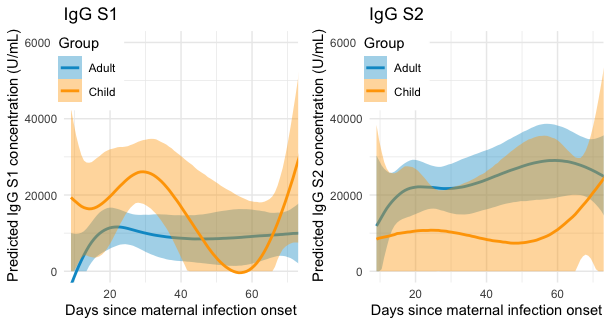
Supplementary Figure 4.** Predicted IgG S1 and S2 concentration (with 95% credible intervals) in combined positive IgG samples from mothers/other household adults and infants/other household children from COVID+/- households (adults n = 28, n_obs_= 78) and children (n = 7, n_obs_= 15) over the two months since maternal infection onset.
